# Supplementary material for: Supramolecular Hydrogen Bonding Assembly from Non-Coplanar Aromatic Tetra-1H-Pyrazoles with Crystallization-Induced Emission (CIE)
Source: Int J Mol Sci. 2022 Apr 11;23(8):4206. doi: 10.3390/ijms23084206 (PMC9026852; doi:10.3390/ijms23084206)
Supplement: Supplementary file 1 [file ijms-23-04206-s001.zip › ijms-1661298-supplementary.pdf]

International Journal of *Molecular Sciences*

Supplementary Materials

Contents

1. Synthesis and calculation
2. X-ray diffraction measurement
3. Powder X-ray diffraction
4. Photoluminescence properties

1. Synthesis and calculation

Materials and Methods

General: All operations were carried out under an argon atmosphere by using standard Schlenk techniques, unless otherwise stated. The starting materials 4-(4,4,5,5-tetramethyl-1,3,2-dioxaborolan-2-yl)-1H-pyrazole, 1,2,4,5-tetrabromobenzene, 1,3,6,8-tetrabromopyrene, 1,1,2,2-tetrakis(4-bromophenyl)ethane and other reagents were obtained as analytical grade from variety commercial providers and used without further purification.

Measurements: ^{1}H NMR and ^{13}C NMR experiments were performed on Bruker Avance DMX400. Elemental analyses (C, H, N) were carried out with a PECHN 2400 analyzer. Column chromatographic separations were carried out on silica gel (200-300 mesh).

Experimental Details

1,2,4,5-tetra(1H-pyrazol-4-yl)benzene (1).

Compound 1 was synthesized via Suzuki coupling reaction. A mixture of n-Butanol/water (180 mL, 3:1 v/v) containing 1,2,4,5-tetrabromobenzene (1.5 g, 3.81 mmol), 1-(tetrahydropyran-2-yl)-4-pyrazoleboronic acid pinacol ester (8.0 g, 28.76 mmol), tetrakis(triphenylphosphine)palladium (0.66 g, 0.29 mmol) and potassium carbonate (3.90 g, 14.29 mmol) was heated at 75-80 °C under stirring for 72 h. The resulting white precipitate was filtered off, dried under vacuum and then purified by column chromatography using ethyl acetate/petroleum ether (15:1) as eluent. 1,2,4,5-tetra(1H-pyrazol-4-yl)benzene (1) was obtained by deprotecting 1,2,4,5-tetrakis(1-(tetrahydro-2H-pyran-2-yl)-1H-pyrazol-4-yl)benzene in methanol (10 mL) and dichloromethane (20 mL) in the presence of 1 M HCl (5 mL) at room temperature. The product precipitated as a white solid which was filtered off and dried under vacuum (yield: 82%). ^{1}H NMR (400 MHz, d_6-DMSO, 298 K) δ: 12.79 (s, 4H), 7.56 (s, 4H), 7.36 (s, 2H), 7.36 (s, 4H). ^{13}C NMR (100 MHz, d_6-DMSO, 298 K) δ: 138.55, 131.778, 130.37, 127.58, 120.65. Elem. Anal. Calcd. for $C_{18}H_{14}N_8$: C, 63.15; H, 4.12; N, 32.73%. Found: C, 63.64; H, 4.02; N, 32.65%.

1,3,6,8-tetra(1H-pyrazol-4-yl)pyrene (2).

Compound 2 was synthesized by performing a Suzuki coupling reactionA mixture of n-Butanol/water (300 mL, 3:1 v/v) containing 1,3,6,8-tetrabromopyrene (3.94 g, 7.62 mmol), 1-(tetrahydro-pyran-2-yl)-4-pyrazoleboronic acid pinacol ester (8.0 g, 28.76 mmol), tetrakis(triphenylphosphine)palladium (0.66 g, 0.29 mmol) and potassium carbonate (3.90 g, 14.29 mmol) was heated at 75-80 °C under stirring for 72 h. The resulting blackish green precipitate was filtered off, dried under vacuum and then purified by column chromatography using ethyl acetate/petroleum ether (15:1) as eluent. 1,3,6,8-tetra(1H-pyrazol-4-yl)pyrene (2), was obtained by deprotecting 1-(tetrahydro-pyran-2-yl)-4-pyrazoleboronic acid pinacol ester in methanol (5mL) and dichloromethane (10 mL) in the presence of 1 M HCl (4 mL) at room temperature. The product precipitated as a green solid which was filtered off and dried under vacuum (yield: 72%). ^{1}H NMR (400 MHz, d_6-DMSO, 298 K) δ: 13.16 (s, 1H), 8.42 (s, 4H), 8.16 (s, 8H), 8.11 (s, 2H). ^{13}C NMR (100 MHz, d_6-DMSO, 298 K)

δ: 130.57, 129.18, 128.80, 127.20, 126.31, 125.02, 120.13. Elem. Anal. Calcd. For $C_{28}H_{18}N_8$: C, 72.09; H, 3.89; N, 24.02%. Found: C, 72.04; H, 3.90; N, 24.05%.

Synthesis of 1,1,2,2-tetrakis(4-(1H-pyrazol-4-yl)phenyl)ethene (3).

Compound 3 was synthesized by performing a Suzuki coupling reaction A mixture of n-Butanol/water (300 mL, 3:1 v/v) containing 1,1,2,2-tetrakis(4-bromophenyl)ethene (4.94 g, 7.62 mmol), 1-(tetrahydro-pyran-2-yl)-4-pyrazoleboronic acid pinacol ester (8.0 g, 28.76 mmol), tetrakis(triphenylphosphine)palladium (0.66 g, 0.28 mmol) and potassium carbonate (3.90 g, 14.29 mmol) was heated at 75-80 °C under stirring for 72 h. The resulting baby blue precipitate was filtered off, dried under vacuum and then purified by column chromatography using ethyl acetate/petroleum ether (15:1) 1,1,2,2-tetrakis(4-(1H-pyrazol-4-yl)phenyl)ethene (3), was obtained by deprotecting 1,1,2,2-tetrakis(4-(1-(tetrahydro-2H-pyran-2-yl)-1H-pyrazol-4-yl)phenyl)ethane in methanol (10 mL) and dichloromethane (20 mL) in the presence of 1 M HCl (6 mL) at room temperature. The product precipitated as a yellowish solid which was filtered off and dried under vacuum (yield: 83%). ^{1}H NMR (400 MHz, d_6-DMSO, 298 K) δ: 12.90 (s, 1H), 8.14 (s, 1H), 7.86 (s, 1H), 7.40 (s, 1H), 6.99 (s, 1H). ^{13}C NMR (100 MHz, d_6-DMSO, 298 K) δ: 141.58, 140.04, 131.86, 131.49, 124.97, 121.27. Elem. Anal. Calcd. For $C_{38}H_{28}N_8$: C, 76.49; H, 4.73; N, 18.78 %. Found: C, 76.44; H, 4.72; N, 18.95%.

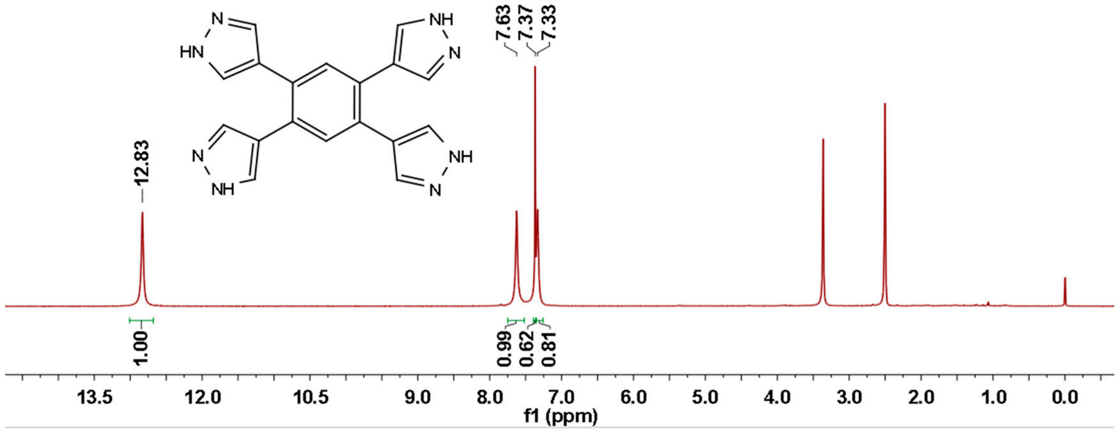

Figure S1. ^{1}H NMR (400 MHz, 298 K) spectrum of compound **1** in d_6-DMSO.

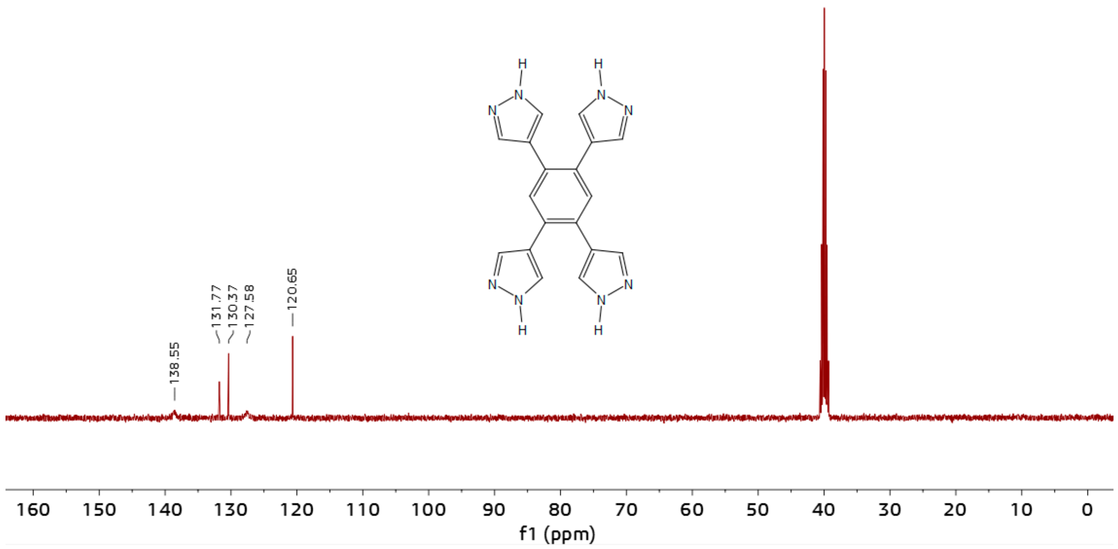

Figure S2. ^{13}C NMR (100 MHz, 298 K) spectrum of compound **1** in d_6-DMSO.

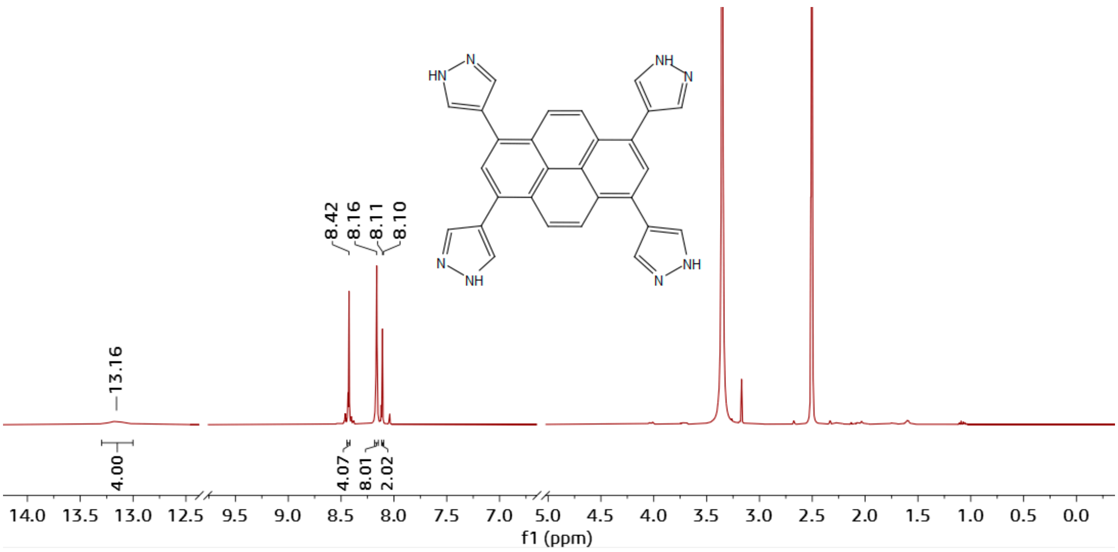

Figure S3. ^{1}H NMR (400 MHz, 298 K) spectrum of compound **2** in d_6-DMSO.

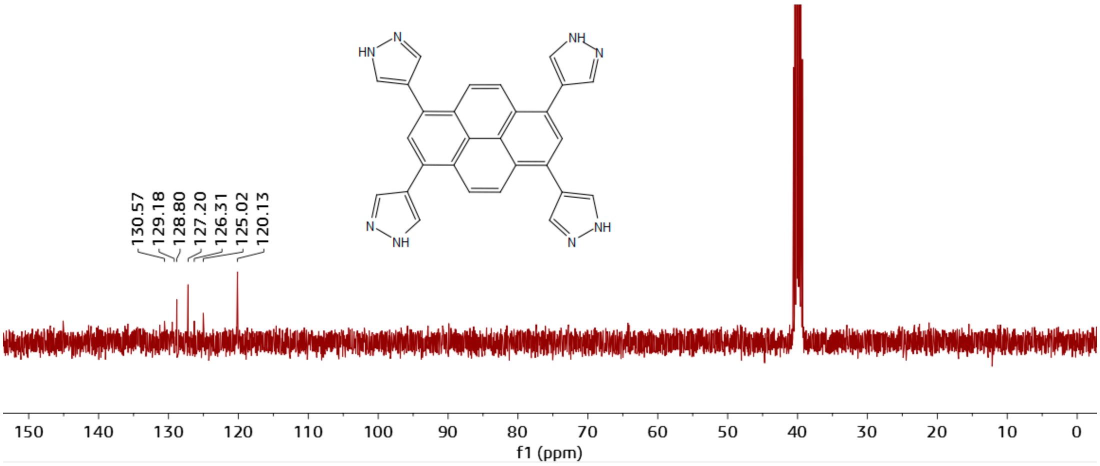

Figure S4. ^{13}C NMR (100 MHz, 298 K) spectrum of compound **2** in d_6-DMSO.

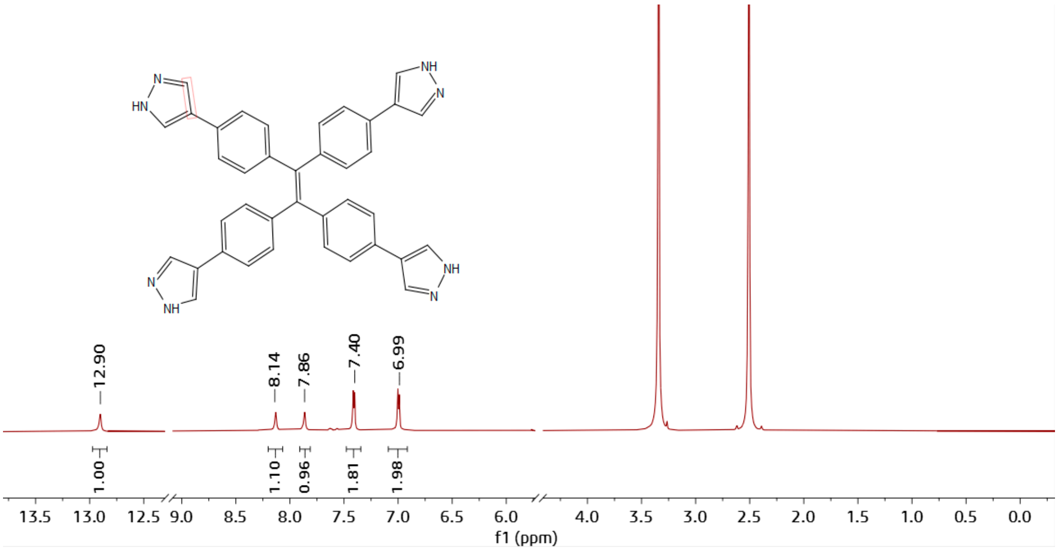

Figure S5. ^{1}H NMR (400 MHz, 298 K) spectrum of compound **3** in d_6-DMSO.

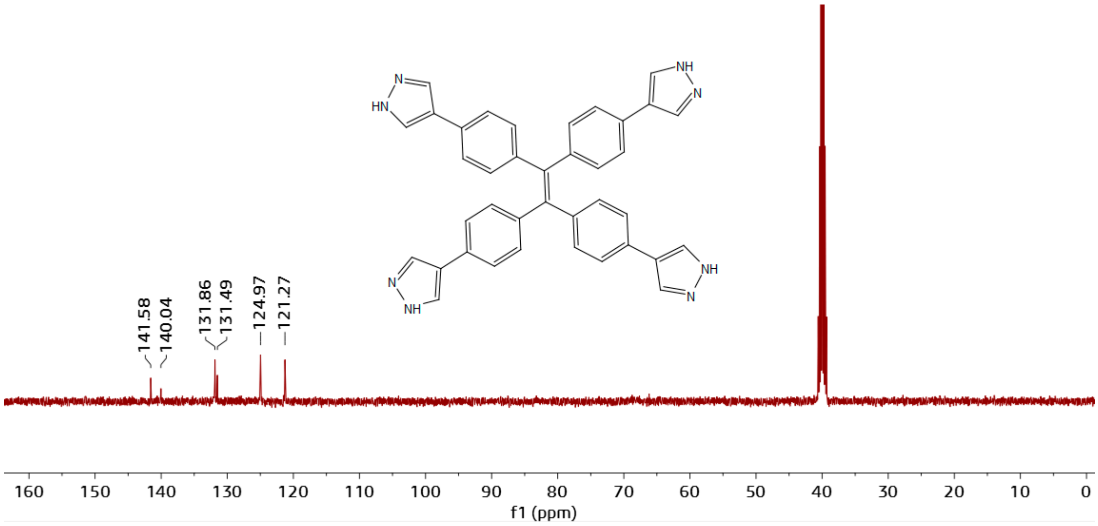

Figure S6. ^{13}C NMR (100 MHz, 298 K) spectrum of **3** in d_6-DMSO.

2. X-ray diffraction measurement

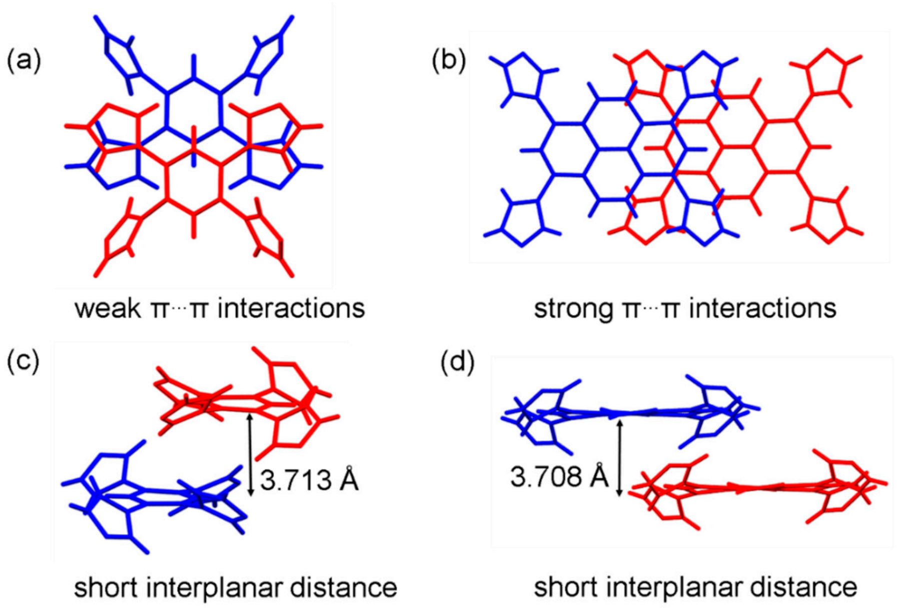

Figure S7. $\pi \ldots \pi$ stacking between adjacent layers observed in structures of **HOF-1** and **HOF-2**.

Table S1. Crystal data and structure refinement for **HOF-1** , **HOF-2,** and **HOF-3**.

	**HOF-1**	**HOF-2**	**HOF-3**
Formula	$C_9H_7N_4$	$C_{14}H_9N_4$	$C38H_{28}N_8$
Formula weight	171.19	233.25	596.68
Temperature/K	293(2)	293(2)	293(2)
Crystal system	monoclinic	orthorhombic	tetragonal
Space group	*C12/c1*	*Pnma*	*P*-42$_1$*c*
a/Å	18.2734(17)	20.7995(16)	15.2584(4)
b/Å	17.8342(19)	22.4471(18)	15.2584(4)
c/Å	8.3083(9)	7.0754(5)	19.5201(12)
α/°	90	90	90
β/°	113.162(3)	90	90
γ/°	90	90	90
Volume/Å^3	2489.4(4)	3303.4(4)	4544.6(4)
Z	8	8	4
ϱ_{calc}g/cm^3	0.914	0.938	0.872
μ/mm^{-1}	0.060	0.059	0.054
F(000)	712	968	1248
Crystal size/mm^3	0.34 × 0.34 × 0.32	0.23 × 0.23 × 0.22	0.34 × 0.32 × 0.30
2Θ range for data collection/°	1.665 – 25.136	1.814 – 23.308	1.694 – 25.100
Index ranges	-21≤h≤21, -21≤k≤18, -9≤l≤9	-23≤h≤22, -24 ≤l≤24, -7≤k≤7	-15≤h≤14, -17≤k≤13, -21 ≤l≤≤23
Reflections collected	14233	25790	22134
Independent reflections [R_{int}]	2217[0.0687]	2449 [0.1069]	4000 [0.0445]
Data/restraints/parameters	22217/0/120	2449/0/163	4000 / 0 / 208
Goodness-of-fit on F^2	1.076	1.171	1.032
*R*1, *wR*2 (*I* > 2(*I*))	0.0674, 0.2067	0.1129, 0.3652	0.0685, 0.2042
*R*1, *wR*2 (all data)	0.0925, 0.2306	0.1445, 0.3912	0..0965, 0.2268
resid. el. dens.[e/Å^3]	-0.474/1.059	-0.473 / 0.571	-0.228 / 0.174
CCDC number	2056570	2056571	2056572

3. Powder X-ray diffraction

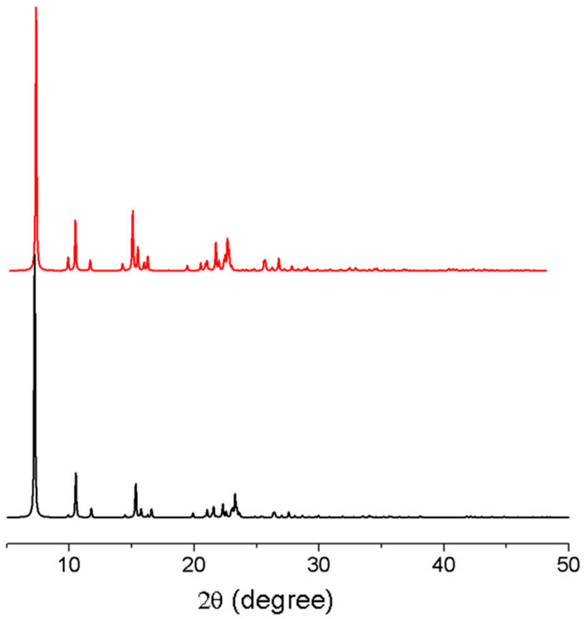

Figure S8. Powder X-ray diffraction (XRD) patterns of as-synthesized and simulated **HOF-1**.

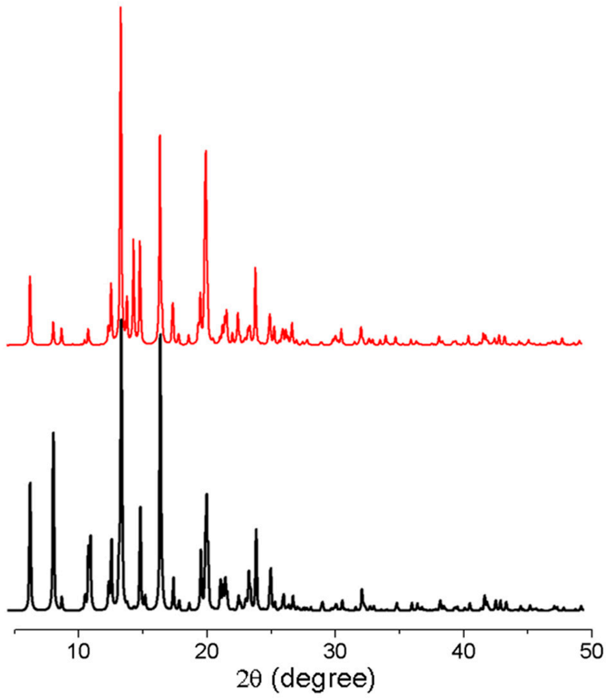

Figure S9. Powder X-ray diffraction (XRD) patterns of as-synthesized and simulated **HOF-2**.

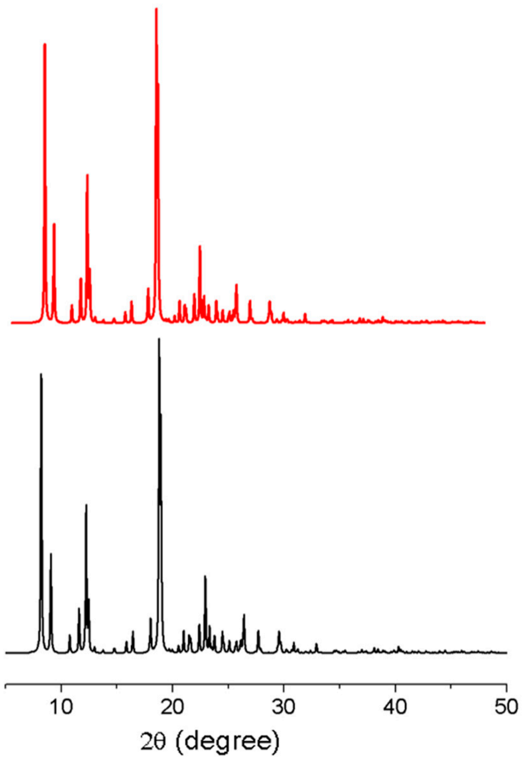

Figure S10. Powder X-ray diffraction (XRD) patterns of as-synthesized and simulated **HOF-3**.

4. **Photoluminescence properties**

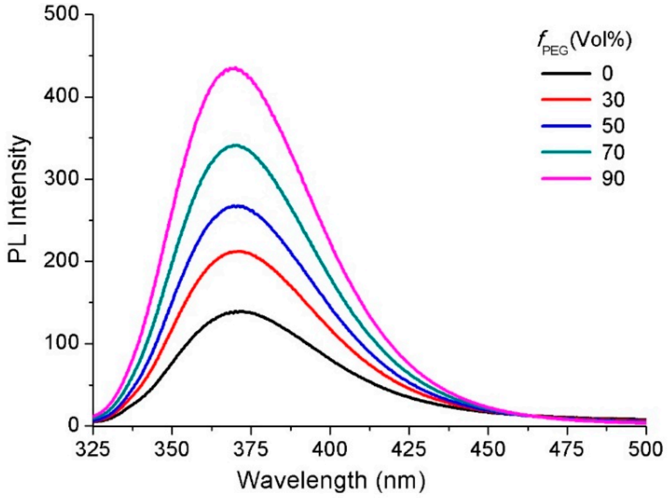

Figure S11. PL spectra of the dilute solutions of **1** ($1.0 \bullet 10^{-5}$ mol L^{-1}) in DMA – PEG1000 mixtures with different volume fractions of PEG1000. Excitation wavelength = 306 nm.

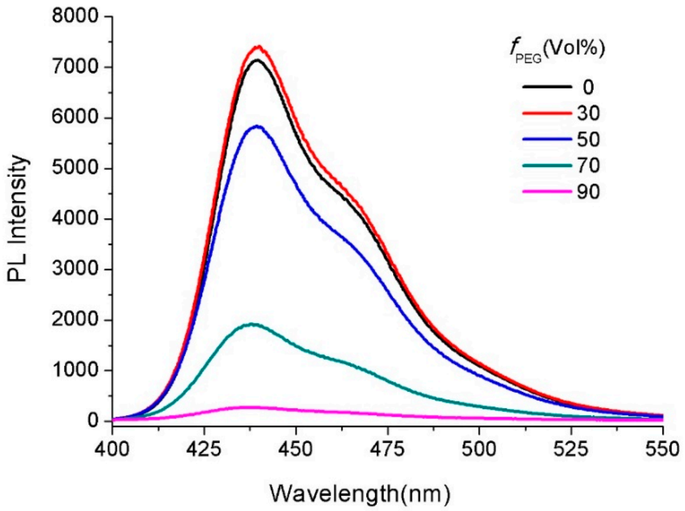

Figure S12. PL spectra of the dilute solutions of **2** ($1.0 \bullet 10^{-5}$ mol L^{-1}) in DMA – PEG1000 mixtures with different volume fractions of PEG1000. Excitation wavelength = 385 nm.

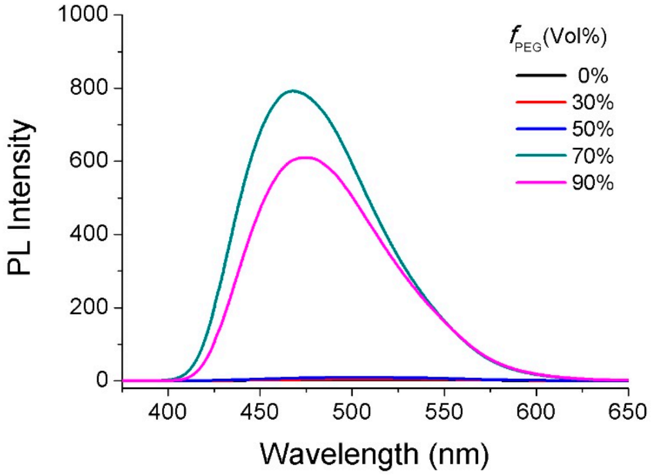

Figure S13. PL spectra of the dilute solutions of **3** ($1.0 \bullet 10^{-5}$ mol L^{-1}) in DMA – PEG1000 mixtures with different volume fractions of PEG1000. Excitation wavelength = 376 nm.
